# Enhanced molecular dynamic simulation studies unravel long-range effects caused by sequence variations and partner binding in RNA aptamers

**DOI:** 10.1016/j.omtn.2023.102039

**Published:** 2023-09-29

**Authors:** Ida Autiero, Luigi Vitagliano

**Affiliations:** 1 Institute of Biostructures and Bioimaging, Via Pietro Castellino 111, 80131 Naples, Italy

**Keywords:** MT: Oligonucleotides: Therapies and Applications, nucleic acids, aptamer, flexibility, RNA conformational distribution, REMD simulation, sequence-structure relationships, structural RNA motifs, plasticity, rational design

## Abstract

Intrinsic flexibility and structural modularity are two common features of RNA molecules. Although functionally crucial, RNA plasticity often represents a major complication in high-resolution structural studies. To overcome this problem, RNAs may be rigidified through the complexation with high-affinity partners such as Fab molecules. This approach has been previously used to characterize the DIR2-aptamer. However, possible perturbations induced by the insertion of the Fab binding site on the DIR2-aptamer conformational properties were not investigated. Here, using enhanced molecular dynamics simulations, we compared the dynamics of the DIR2 aptamer holding the Fab binding site with that of the parental sequence. Our results suggest that the L2-loop modification for the Fab recognition leads to a significant increase in local flexibility that also affects the mobility of distant regions. The trajectories provide clear indications of the groups and the interactions mediating the dynamics transfer in DIR2. The effectiveness of our approach in addressing RNA flexibility was further corroborated by showing its ability to reproduce the most important events affecting the NF-κB RNA aptamer upon dissociation from the partner. Therefore, REMD analyses, a rarely adopted technique to unravel the structural/dynamical properties of aptamers, could efficiently complement experimental data guiding the rational design of nucleic acid therapeutics.

## Graphical abstract

**Figure:**
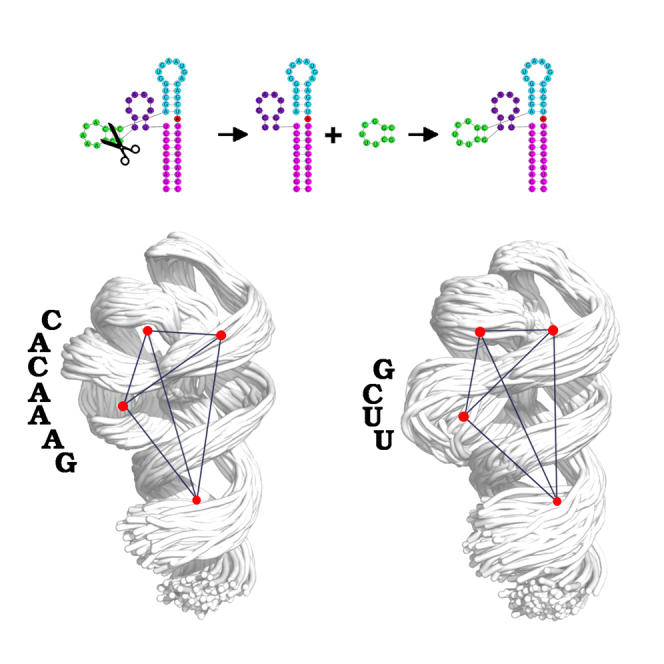

## Teaser

The RNA plasticity allows a huge functional versatility but hampers the characterization. We provide a thorough picture of the structural and dynamic consequences caused by sequence modifications for an RNA aptamer. Our approach of modeling and theoretical simulations represents an ideal method for the rational design of nucleic acid therapeutics.

## Introduction

RNA has fundamental roles in life, as it regulates a plethora of cellular functions.^1^^,^^2^ The intrinsic structural versatility of RNA makes it able to efficiently target diverse molecules. Therefore, it is not surprising that the RNA derivatives represent promising systems for a wide range of biomedical applications.^3^⁠ Since the 90s, the discovery of new RNAs targeting specific proteins and other molecules of biological interest was made possible by the development of the systematic evolution of ligands by exponential enrichment (SELEX) approach.^4^⁠ SELEX consists of cyclic processes of identification and amplification of RNA/DNA molecules among a pool of random sequences that were selected on their ability to bind the target of interest. The potentiality for a wide range of applications and the good efficacy in their selection gave rise to a rapid growth of interest in RNAs.^5^^,^^6^ However, a limiting factor for the development of these molecules for biomedical applications is the paucity of structural information available as atomic-level characterizations of RNAs are quite difficult and time-demanding due to their intrinsic flexibility.^7^^,^^8^ In 2019, the standalone RNA molecules represent only 1% of the structures deposited in the Protein DataBank (PDB) and the number of RNA aptamers in complex with the protein target is as low as 63 as highlighted by a recent search of PDB (www.rcsb.org) (March 2023). The relative scarcity of structural information strongly limits our understanding of the RNA folding process as well as of its ability to adopt specific three-dimensional conformations that are frequently able to establish high-affinity interactions with diversified partners. One effective and used strategy to overcome this general issue is based on the structural characterization of aptamers following their complexation to high-affinity Fabs, by adapting a similar approach used for highly flexible proteins.^9^^,^^10^^⁠^ In the case of aptamers, this procedure involves the modification of the aptamer sequence by the insertion of a structural motif that is specifically recognized by a Fab that operates as a crystallization chaperone.^11^

This strategy has been successfully employed to structurally characterize DIR2, a fluorogenic RNA aptamer endowed with important potentialities in imaging techniques since it is able to activate fluorescence upon binding chromophore molecules. Indeed, DIR2 binds the chromophores Dimethylindole red (DIR) and oxazole thiazole blue (OTB) with nanomolar affinity, thus inducing the emission of red and blue fluorescence, respectively. The intrinsic flexibility of DIR2 impaired its ability to crystallize and perform high-resolution crystallographic studies.^11^ A variant of the aptamer was generated by replacing its UUCG loop with an AAACA pentaloop closed by a G-C pair that is a recognition motif for the Fab BL3-6. The ability of the Fab BL3-6 to act as a crystallization chaperone and to reduce the flexibility of the aptamer allowed the crystallographic characterization of the Fab BL3-6-DIR2 complex, also in combination with the OTBSO3 fluorophore. These investigations unraveled that, in contrast to other fluorogenic aptamers that typically exhibit G-quadruplex- or nucleobase tetrad-like motifs, DIR2 aptamer adopts a compact, tuning fork-like structure composed of a helix and two short stem-loops that generate the ligand binding site through a network of tertiary interactions.^11^⁠ Although the authors clearly showed that the insertion of the Fab recognition loop does not have important effects on the fluorescence emission and presumably does not affect the global structure of the aptamer, the intrinsic dynamic properties of this system and how they are affected by the insertion of the loop and/or by its binding to the Fab remain uncharacterized. In this scenario, we here investigated the intrinsic structural and dynamic properties of the wild-type DIR2 (DIR2wt) and of its variant with a high affinity for Fab BL3-6 (DIR2Ffab), by performing extended conformational samplings by using the replica exchange molecular dynamics (REMD) method. REMD is an enhanced sampling approach of molecular dynamic simulation that allows the overcoming of high-energy barriers present in the conformational space.^12^⁠

Moreover, we also show that the method here illustrated can be reliably applied to gain insights into the structural basis of the aptamer-protein recognition process. In particular, using the well-characterized interaction between an RNA aptamer of the nuclear factor (NF)-κB transcription factor,^13^^,^^14^ we show the effectiveness of the present approach in predicting the main structural and dynamic events associated with the dissociation of the aptamer from the protein partner.

## Results

### Static three-dimensional models of the aptamers and their general structural properties

**Figure 1:**
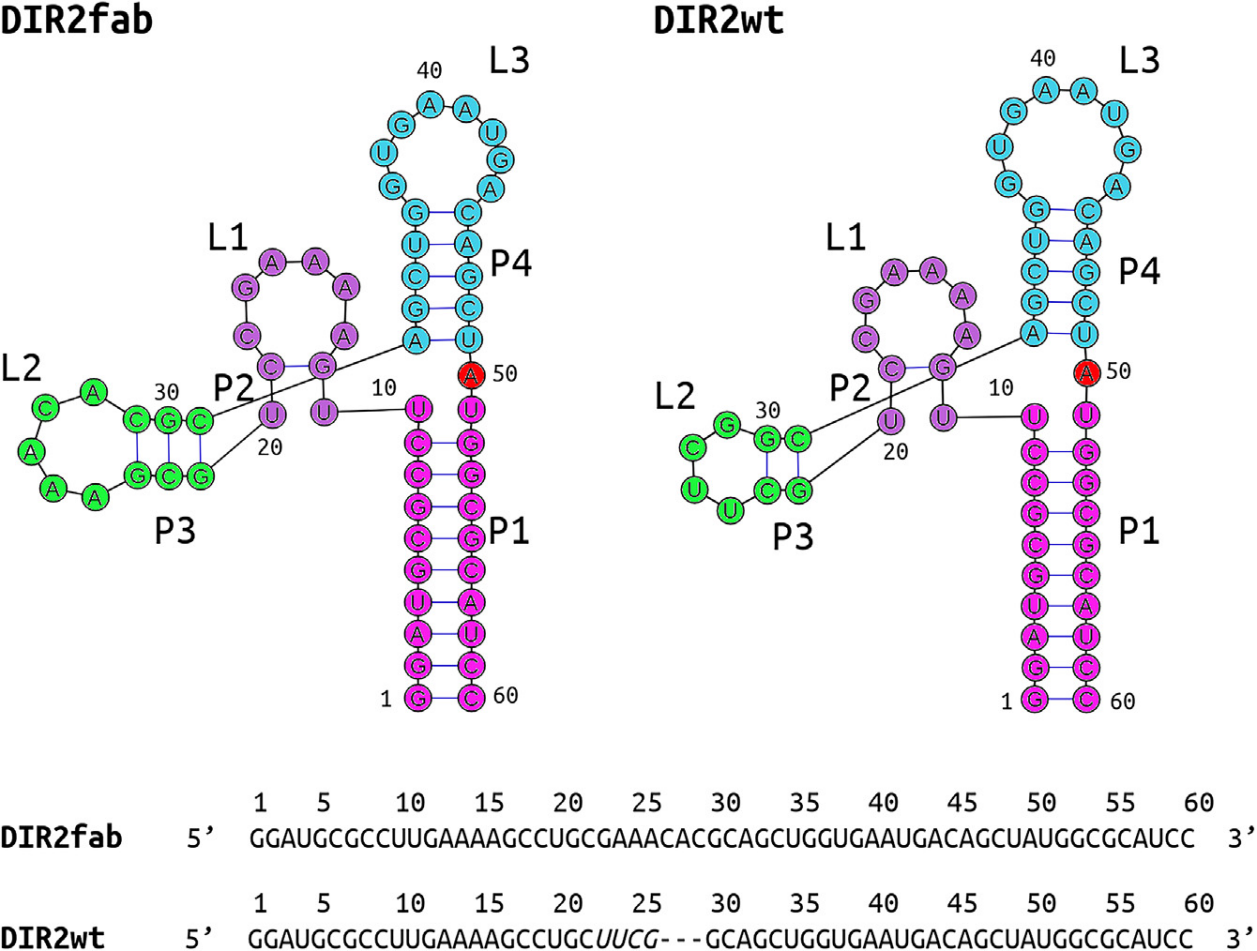
The secondary structure and sequence of the DIR2fab and DIR2wt aptamers The loop that received the sequence replacement is indicated in italic in the DIR2wt sequence.

The intrinsic dynamic and structural properties of the DIR2 aptamer and the impact that the insertion of the Fab recognition motif has on its properties were here investigated by performing REMD simulations to provide an enhanced sampling of the conformational ensemble of these intricate biomolecules. As detailed in the materials and methods section, the coordinates of the aptamer containing the insertion of the Fab-loop motif (DIR2fab) were extracted from the structure of the complex of this variant with Fab BL3-6 (PDB entry 6db9^11^). On the other hand, for the wild-type DIR2 aptamer (DIR2wt) for which no experimental structure is currently available, a reliable three-dimensional model was generated using the structure of DIR2fab as template and manually replacing the Fab-loop motif (GAAACAC) with the parental UUCG sequence (Figure 1). The model of DIR2wt is composed of four helices P1, P2, P3, and P4, and three loops L1, L2 and L3 (Figure 1). The initial P1 helix is followed by the coaxial P4-L3 stem-loop, which arranges into a tuning fork-like fold, similar to P2-L1. P4-L3 and P2-L1 are connected essentially by Watson-Crick connections, while non-canonical interactions stabilize L1-L3 proximity in a precise tertiary fold. The loop L2 contains UUCG that is replaced by the Fab BL3-6 binding site in DIR2fab (Figure 1). The DIR2fab presents the same fold and, therefore, embodies the same structure elements. The nucleotides of the sequences of the two aptamers were denoted using the same numeration scheme, which was adapted from Piccirilli et al. for DIR2fab, as a consequence the shorter DIR2wt lacks the bases numbered 27, 28, and 29.

### Overall analysis of the REMD simulations

**Figure 2:**
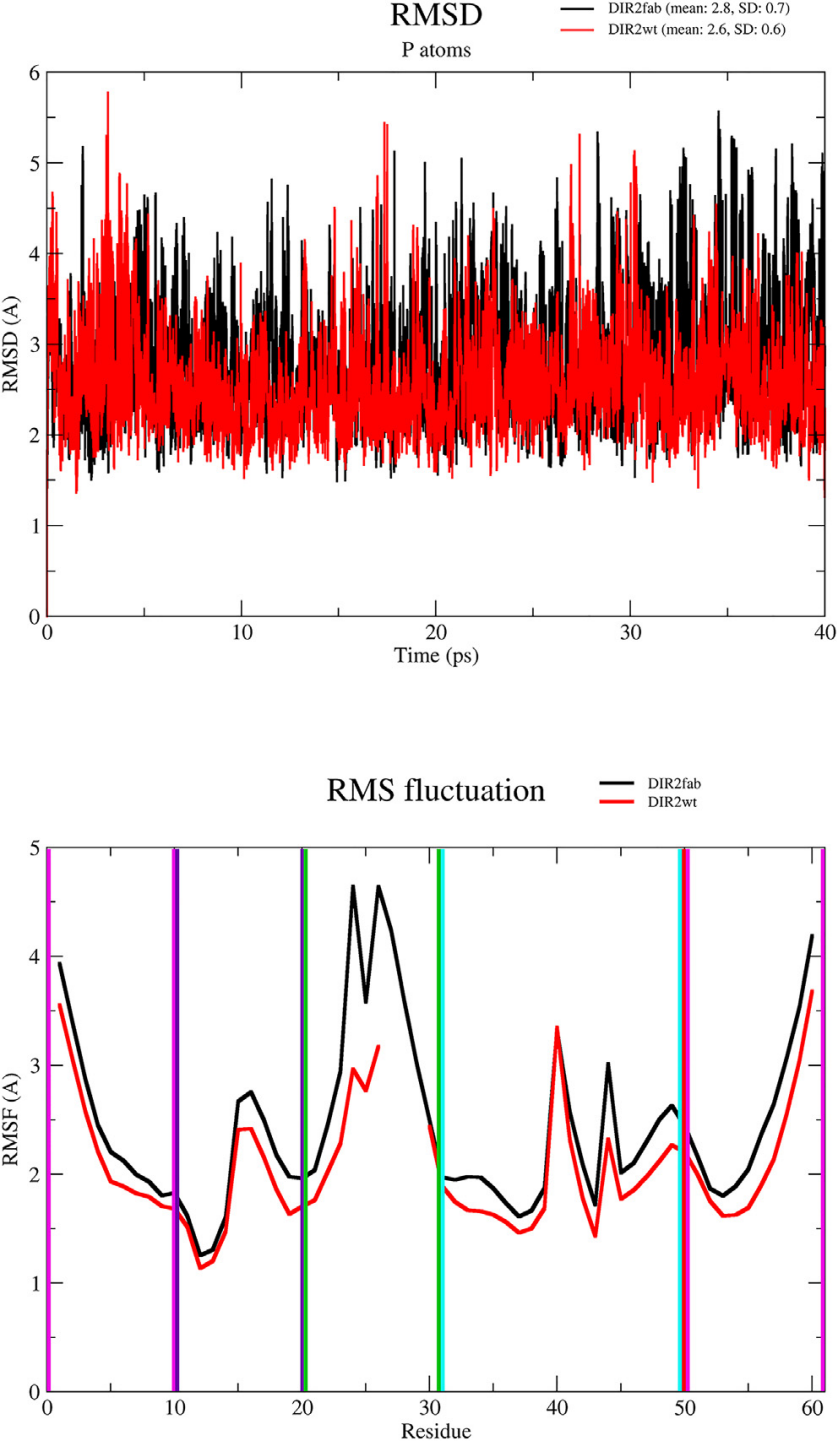
RMSD and RMSF profiles Top: The RMSD values of trajectory structures vs. the starting model (black: DIR2fab and red: DIR2wt). Bottom: The RMSF profiles of trajectory structures along the simulations (black: DIR2fab and red: DIR2wt). The residues belonging to the secondary structure motifs are highlighted with colored boxes using the following color scheme: P1: magenta, P2L1: violet, P3L2: green, and P4L3: cyan.

Following the common protocol of the REMD methodology, 64 independent simulations were performed in the temperature range 300–385 K (see materials and methods for details). One important aspect for the successful application of this approach is the exchange rate of replica between different temperature values. For DIR2wt and DIR2fab, the average probabilities were 0.30 and 0.32, respectively. These values indicate that for both DIR2wt and DIR2fab, a satisfactory exchange rate was achieved. All analyses were performed on the ensemble of structures collected at 300 K. As shown in Figure 2 (top panel), the inspection of the root-mean-square deviation (RMSD) values of the trajectory models computed against the corresponding starting run structure indicates that for the two forms of the DIR2 aptamer, the structures that emerged from the simulations present similar deviations from the respective starting models. The inspection of the RMSD values also highlights that they significantly oscillate throughout the trajectories, thus indicating that both aptamers are endowed with a remarkable level of structural flexibility. This finding fully agrees with the reported failure to grow crystals of these aptamers^11^ and with the necessity to resort to the Fab complexation to gain structural information on DIR2. To gain further insights into the intrinsic flexibility of DIR2fab and DIR2wt, we then evaluated the root-mean-square fluctuations (RMSF) *per* residues that were computed on the entire 40 ns of REMD simulation time (Figure 2, bottom panel). As expected, terminal regions of the two aptamers present higher RMSF values. High RMSF values are also displayed by the nucleotides of the L2 loop. It is important to note that, compared with DIR2wt, the L2 loop of the DIR2fab variant presents remarkably higher flexibility due to the insertion of the Fab binding motif (residues 23–29). In DIR2fab, these residues, which interact with the Fab molecule in the X-ray structure of the complex (Figure S1), make only sporadic connections to other residues of the same region G23-C29, A24-A25, and A25-A28 (see Table 1). Interestingly, the different interaction pattern that the L2 loop establishes with the rest of the molecule has a remarkable impact on the overall flexibility of the two aptamers. Indeed, as shown in Figure 2, DIR2fab is endowed with generally larger fluctuations compared with DIR2wt, even in regions that are distant from the inserted Fab binding motif. This observation suggests that the introduction of this motif may have an impact on the overall flexibility of the aptamer. Nevertheless, it is important to note that the flexibility of the residues within the L3 region of the aptamers that is deputed to the binding of the fluorophores (nucleotides 39–42) is not remarkably different in the two variants. This is in line with the experimental evidence of a similar binding of the fluorophores exhibited by DIR2fab and DIR2wt.^11^

**Table 1:** The percentage of occurrence of persistent (>10% of the run frames) hydrogen bonds along the DIR2fab and DIR2wt simulations are reported

Hydrogen bond pairs	DIR2fab	DIR2wt
**P1**		

G1(O6)-C60(N4)	39.1	39.3
G1(N2)-C60(O2)	49.4	51.1
G1(N1)-C60(N3)	53.0	53.4
G2(O6)-C59(N4)	40.8	40.2
G2(N1)-C59(N3)	60.9	59.6
G2(N2)-C59(O2)	61.9	61.1
A3(N6)-U58(O4)	24.1	24.3
A3(N1)-U58(N3)	43.2	45.3
U4(O4)-A57(N6)	35.6	34.3
U4(N3)-A57(N1)	48.2	48.8
G5(O6)-C56(N4)	43.2	43.8
G5(N1)-C56(N3)	58.1	59.8
G5(N2)-C56(O2)	61.2	60.5
C6(N4)-G55(O6)	42.2	42.4
C6(N3)-G55(N1)	57.6	58.6
C6(O2)-G55(N2)	59.8	60.7
G7(N2)-C54(O2)	61.4	62.5
G7(O6)-C54(N4)	43.2	43
G7(N1)-C54(N3)	62.4	62.1
C8(N4)-G53(O6)	44.0	45.9
C8(N3)-G53(N1)	56.1	55.7
C8(O2)-G53(N2)	62.1	60.7
C9(N4)-G52(O6)	43.4	42.1
C9(N3)-G52(N1)	63.7	64.2
C9(O2)-G52(N2)	65.8	65.6
U10(O2)-U51(N3)	30.7	29
U10(N3)-U51(O4)	39.5	41.5

**P2L1**		

**U11(O4)-U20(N3)**		**11.8**
*∗U11( O 2P)-C31( O 2′)*	*12.2*	*12*
U11(N3)-U20(O4)	12.8	13.6
U11(N3)-U20(O2)	13.9	19.5
U11(O2)-U20(N3)	36.3	32.5
***G12(O2′)-A13(O1P)***	***11.0***	
*∗G12( O 2′)-C45( O 2P)*	*22.5*	*26.6*
G12(O6)-C19(N4)	49.3	49.2
G12(N1)-C19(N3)	58.6	57.1
G12(N2)-C19(O2)	65.6	62.5
***∗A13(O1P)-G37(N2)***	***10.6***	
*∗A13( O 1P)-G37(N1)*	*13.4*	*12.5*
*∗A13( O 2P)-C45(N4)*	*14.8*	*13.4*
***∗A13(O1P)-C45(N4)***		***10.5***
*∗A13( O 2′)-G43(N7)*	*36.4*	*38.5*
∗A14(N6)-U42(O2)	30.3	30.2
∗A14(N1)-U38(N3)	38.1	38.8
∗A14(N6)-U38(O2)	46.8	46
∗A14(N7)-U42(N3)	48.2	47.2
∗A15(N6)-U42(O4)	15.9	15.2
*∗A15(N6)-A41( O 2′)*	*16.9*	*16.7*
∗C18(N4)-G43(O6)	41.4	41.3
∗C18(N3)-G43(N1)	55.9	53.5
∗C18(O2)-G43(N2)	61.5	61.2

**P3L2**		

G21(O6)-C31(N4)	37.0	37.1
G21(N1)-C31(N3)	50.4	51.6
G21(N2)-C31(O2)	56.9	57.5
***C22(N4)-C27(O1P)***	***11.7***	
C22(N4)-G30(O6)	45.0	49.4
C22(N3)-G30(N1)	60.0	61.8
C22(O2)-G30(N2)	62.9	64.1
**G23(O6)-C29(N4)**	**41.4**	
**G23(N2)-C29(O2)**	**52.1**	
**G23(N1)-C29(N3)**	**52.1**	
***U23(O2′)-U24(O5′)***		***16.8***
***U23(O2′)-G26(O6)***		***26***
***U23(O2)-G26(N1)***		***31.6***
***A24(O1P)-A25(N6)***	***17.3***	
**∗U24(O4)-G52(N2)**		**33.1**
***U24(O2P)-C25(N4)***		***36.8***
**A25(N3)-A28(N6)**	**23.2**	
***C25(O2′)-G26(O2P)***		***28.5***
***G26(O2′)-G30(O1P)***		***22.8***

**P4L3**		

*C31( O 2′)-A32( O 2P)*	*16.9*	*15.9*
A32(N6)-U49(O4)	34.4	33.3
A32(N1)-U49(N3)	49.1	48.3
G33(O6)-C48(N4)	43.0	42.2
G33(N1)-C48(N3)	58.2	58.1
G33(N2)-C48(O2)	65.5	63.6
C34(N4)-G47(O6)	43.9	45
C34(N3)-G47(N1)	58.6	59.4
C34(O2)-G47(N2)	63.4	65.1
U35(O4)-A46(N6)	42.8	44.1
U35(N3)-A46(N1)	44.2	44.8
G36(O6)-C45(N4)	46.2	48.4
G36(N2)-C45(O2)	59.0	60.5
G36(N1)-C45(N3)	60.8	62.8
G37(N2)-A44(N3)	15.6	19.1
*G37( O 2′)-A44(N1)*	*28.8*	*34.1*
*G39( O 2′)-A41(N3)*	*17.6*	*17.5*
G39(N2)-A41(N1)	50.3	50.6
*A41( O 2′)-U42( O 2P)*	32.2	29

The pairs are divided into sections following the scheme of secondary structure motifs, magenta: P1, P2L1, P3L2, and P4L2. Those connections involving two different motifs are highlighted with an asterisk. The backbone hydrogen bonds are in italics and those differentiating the DIR2fab and DIR2wt aptamer simulations are in italics and bold .

### Intra-motif hydrogen bonds

**Figure 3:**
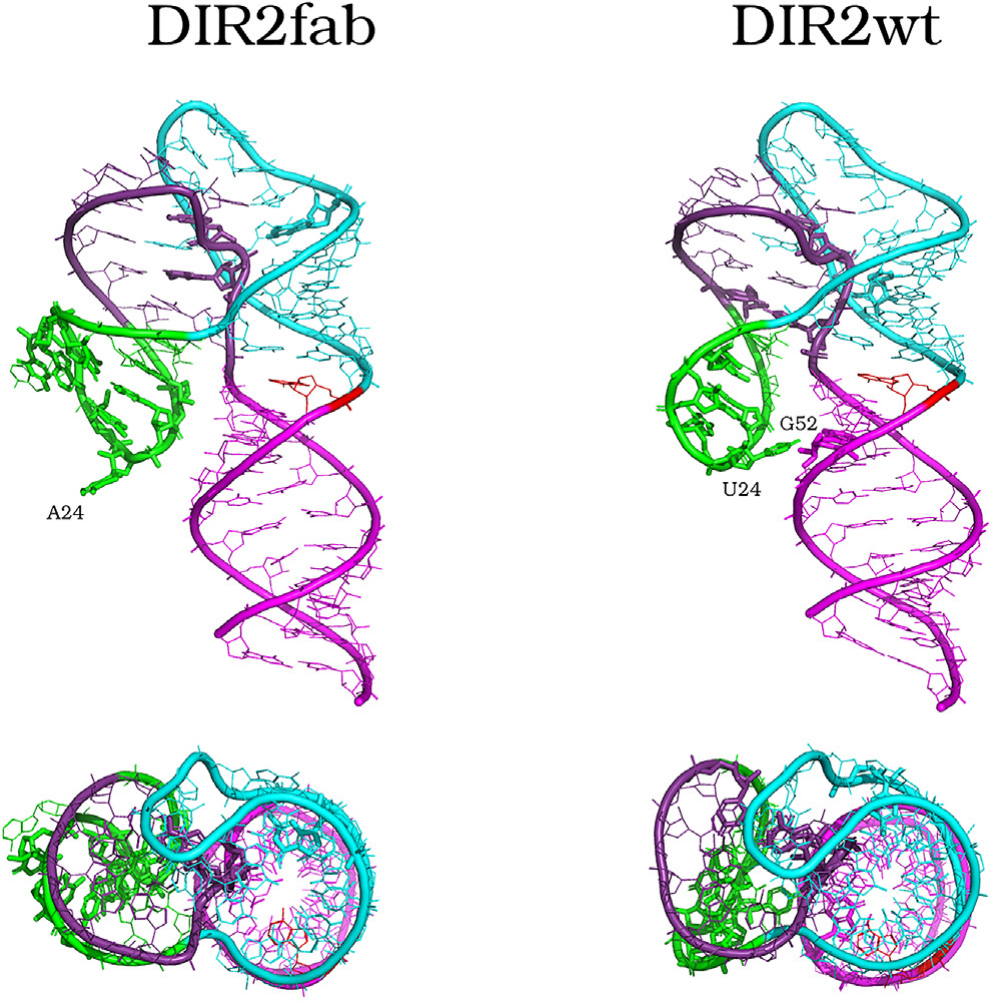
Hydrogen bond network differentiating and peculiar of the two aptamers Cartoon and lines representation of the DIR2fab (left) and DIR2wt (right) representative MD structures. In sticks are shown those residues involved in hydrogen bonds differentiating the DIR2fab compared with the DIR2wt parental sequence.

The intriguing distinct overall flexibility of the two DIR2 variants prompted us to perform a detailed analysis of the evolution of the hydrogen bond networks that stabilize their folding (Tables 1 and 2, Figure S1). This analysis indicates that the crystallographic hydrogen bonds holding the P1 helix base pairs are quite stable in both DIR2fab and DIR2wt runs, in a comparable fashion. Similarly, in both systems the P4-L3 stem-loop maintains most of the crystallographic hydrogen bonds, yet in DIR2wt run a persevered connection between G37 and A44 residues is slightly more stable than in the DIR2fab. Further, in the P2-L1 stem-loop, the U11 residue connects to U20 in both the aptamers, but the G12-A13 connection is peculiar to DIR2fab, in this motif. Precisely, in DIR2fab A13 interacts with either G12 and G37 of P4 and to a lesser extent with C45 of P4 helix, while in DIR2wt A13 interacts in minor measure with G37 but more firmly with C45 (Tables 1 and 2). Because of the lack of the residues 26 to 29 in DIR2wt aptamer sequences and the loop sequence differences, the P3-L2 shows a higher degree of HB network differences among the two simulations; indeed, this stem-loop maintains the initial fold by a completely different HB network. G21-C31 and C22-G30 are persistently connected in both variants. On the contrary, C22-C27, G23-C29, A24-A25, and A25-A28 are peculiar hydrogen bonds of the DIR2fab aptamer, whereas U23-U24, U23-G26, U24-G52, U24-C25, C25-G26, and G26-G30 interact exclusively in the DIR2wt. Notably, the G23-C29 pair of DIR2fab is significantly more persistent than the equivalent U23-G26 of DIR2wt, in terms of connection persistence along the simulations (see HB occurrence, Table 1, Figure 3), because in this aptamer the same residue connects also to U24. The P4L3 region shows a similar dynamic behavior in DIR2fab and DIR2wt. The persistent hydrogen bond connections differentiating the two systems and peculiarly expressed in one of them, are summarized in Table 2 and shown in Figure 3, where DIR2fab and DIR2wt representative conformations extracted from the relative REMD trajectories by the clustering method (see the materials and methods section) are reported.

**Table 2:** The percentage of occurrence of hydrogen bonds differentiating the DIR2fab and DIR2wt simulations is reported

Different hydrogen bonds among the two variants		
Hydrogen bond pairs	DIR2fab	DIR2wt
**U11(O4)-U20(N3)**		11.8
***G12(O2′)-A13(O1P)***	11.02	
***∗A13(O1P)-G37(N2)***	10.55	
***∗A13(O1P)-C45(N4)***		10.5
***C22(N4)-C27(O1P)***	11.72	
**G23(O6)-C29(N4)**	41.36	
**G23(N2)-C29(O2)**	52.11	
**G23(N1)-C29(N3)**	52.14	
***U23(O2′)-U24(O5′)***		16.8
***U23(O2′)-G26(O6)***		26
**U23(O2)-G26(N1)**		31.6
***A24(O1P)-A25(N6)***	17.32	
**∗U24(O4)-G52(N2)**		33.1
***U24(O2P)-C25(N4)***		36.8
**A25(N3)-A28(N6)**	23.19	
***C25(O2′)-G26(O2P)***		28.5
***G26(O2′)-G30(O1P)***		22.8

In bold, the peculiar hydrogen bonds, present only in one of the two variants; italics indicates hydrogen bond involving backbone atoms. in bold and italics hydrogen bonds involving backbone atoms and peculiarly present in only one of the two aptamer variants. The pairs are reported following the scheme of secondary structure motifs, P1, P2L1, P3L2, and P4L2. Those connections involving two different motifs are highlighted with an asterisks.

### Inter-motif connections

The analysis of the relative orientation along the trajectories of different secondary structure motifs is a measure of the plasticity and compactness of the systems. For the two simulations, we calculated the distances between the centers of mass of each motif (Figure S3). Similarly, the P1 helix is closed to the P2L1 and P4L3 stem-loops (black and green lines, Figure S3) and P4L3 to P2L1 and P3L2 (yellow and brown, respectively, Figure S3). P3L2 shows the same distance pick values from P1 and P4L3 in DIR2wt (red and brown, Figure S3), but the P1-P3L2 distance results are wide and higher in DIR2fab (Figure S3). Additionally, P1 and P3L2 approach each other in slightly different behavior, with distance peaks around 25 and 21 Å, in DIR2fab and DIR2wt, respectively (red, Figure S3). These data are consistent with the HB network analyses as the approaching of P1 to P3L2 motifs is favored by the interaction between U24 in L2 and G52 in P1 of DIR2wt. The P1-P3L2 approach is consistent with the DIR2 available crystallographic data on DIR, as in the fluorophore-bound form (PDB code 6DB8) these two secondary elements are closer than in ligand-free form (PDB code 6DB9). Nevertheless, the differences in the relative motif distances between the two aptamer variants do not impact the intrinsic flexibility of the region deputed to bind the fluorophore ligand, that corresponds to the L3 loop (Figure 2, bottom panel, residues 39–40). To assess the impact of the loop replacement in the conformation prone to bind the fluorophore molecules, we also monitored the flip rotation of the glycosidic angles of A40 and G39, which are the main residues involved in fluorophore binding, along the simulations (Figure S4). For both the aptamer variants, this analysis suggests that the G39 shows a narrower distribution of the glycosidic angle if compared with the A40 which shows a larger distribution of the same angle values and thus a wide range of rotational events. Further, the distances of the center of mass of the G39 and A40 bases compared with the previous and the succeeding residues (38–39, 39–40, and 40–41 pairs, Figure S5) suggest that the A40 is the residue with elevated mobility, in line with its high RMSF value (Figure 2). Indeed, this is the key residue prone to flip out from the loop favoring the accommodation of the fluorophore.

In summary, the global RNA architecture is comparably maintained in the two aptamers along the simulations, but the L2 loop interacts more significantly with the P1 helix in DIRwt, as in DIR2fab this tertiary connection is lost and the L2 dynamic is completely independent from the remaining aptamer structure (Figure 3).

### The structural transition of an NF-κB-binding aptamer from the bound to the free state

**Figure 4:**
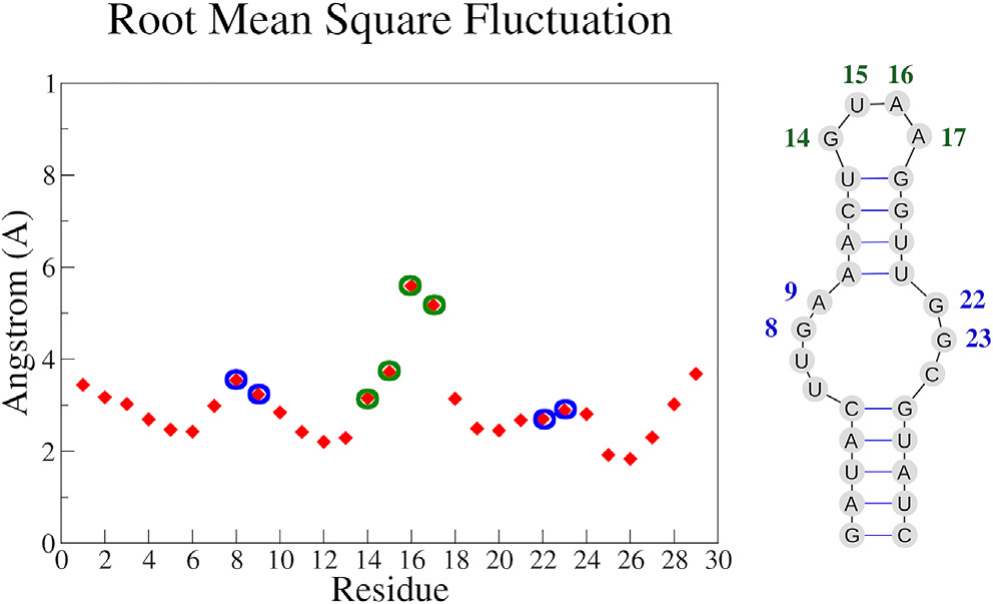
RMSF and secondary struture of NF-kB aptamer Left: The RMSF profiles of REMD NF-κB trajectory structures along the simulations. The residues belonging to the tetraloop (green) and internal (blue) loops are highlighted with colored circles. Right: Secondary structure of the NF-κB aptamer with the indication of the tetraloop and internal loop residues.

**Figure 5:**
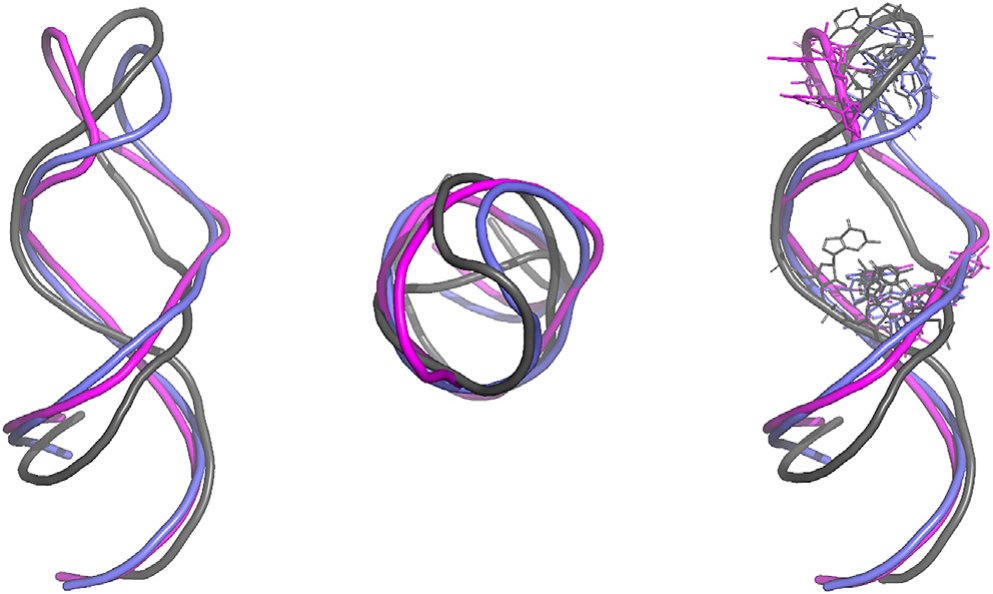
NF-kB aptamer Frontal and top views of the superimposition of the representative REMD NF-κB aptamer conformation (magenta) on the free (violet) and bound (gray) aptamer state, as solved with X-ray and NMR methods (PDB codes: 2JWV and 1OOA, respectively^13^^,^^14^). The tetraloop (14–17) and the internal loop (8, 9, 22, and 23) residues are shown in sticks in the right panel.

The ability of the approach here presented to unravel the long-range effects in the structure of the DIR2 induced by sequence modifications prompted us to check whether this method could be effectively exploited to gain information in the protein-aptamer recognition process. In detail, we investigated the intrinsic structural and dynamic properties of an RNA aptamer specifically selected to target the transcription factor NF-κB (Figure 4).^13^^,^^14^ Extensive experimental studies have demonstrated that the structure of this aptamer undergoes significant modification upon binding to the target protein.^25^ In particular, the comparison of the ligand-free structure of this aptamer determined by NMR with that found in the crystalline structure of the complex with NF-κB highlights clear variations in the tetraloop and the internal loop regions (see Figures 4 and S7).^13^^,^^14^ According to the authors, in this case, protein-aptamer recognition relies on the delicate balance between the structural preorganization of the aptamer and the induced fit caused by the binding.^13^ To check the ability of our approach to predict the structural variation of the aptamer coupled with its dissociation from the transcription factor, we performed REMD simulation starting from the state it assumes in the complex with the protein and compared the trajectory structures with both the free and bound state experimentally observed. The inspection of the RMSF values of the aptamer clearly indicates that different regions of this RNA molecule are endowed with different flexibility. In line with the available experimental data, the tetraloop region (residues 14–17) is characterized by remarkable mobility (Figure 4). The trajectory structures were then clustered as reported in the materials and methods sections. The representative example of the most populated cluster was then compared with the free and the bound states of the aptamer (Figure 5). As shown in Figure 5, the structure of the internal loop region that emerged from the REMD closely resembles the experimental free state, although the bound state was used as the starting model. Notably, in the trajectory structures, some of the distances between bases that characterize the free state are frequently detected. For example, the distances of the pair of bases G8(N9)-G23(N9) and A9(N9)-G23(N9) detected in the simulation are closer to those experimentally observed in the free rather than the bound state (Figure S8). Moreover, in some structures of the trajectory, the G23 base exhibits the swing motion that is associated with the aptamer relaxation following the dissociation from the protein partner (Figures 4 and S8), mainly resulting in an opening of the internal loop region, thus a higher distance of G23 versus G8 as well as A9.

**Figure 6:**
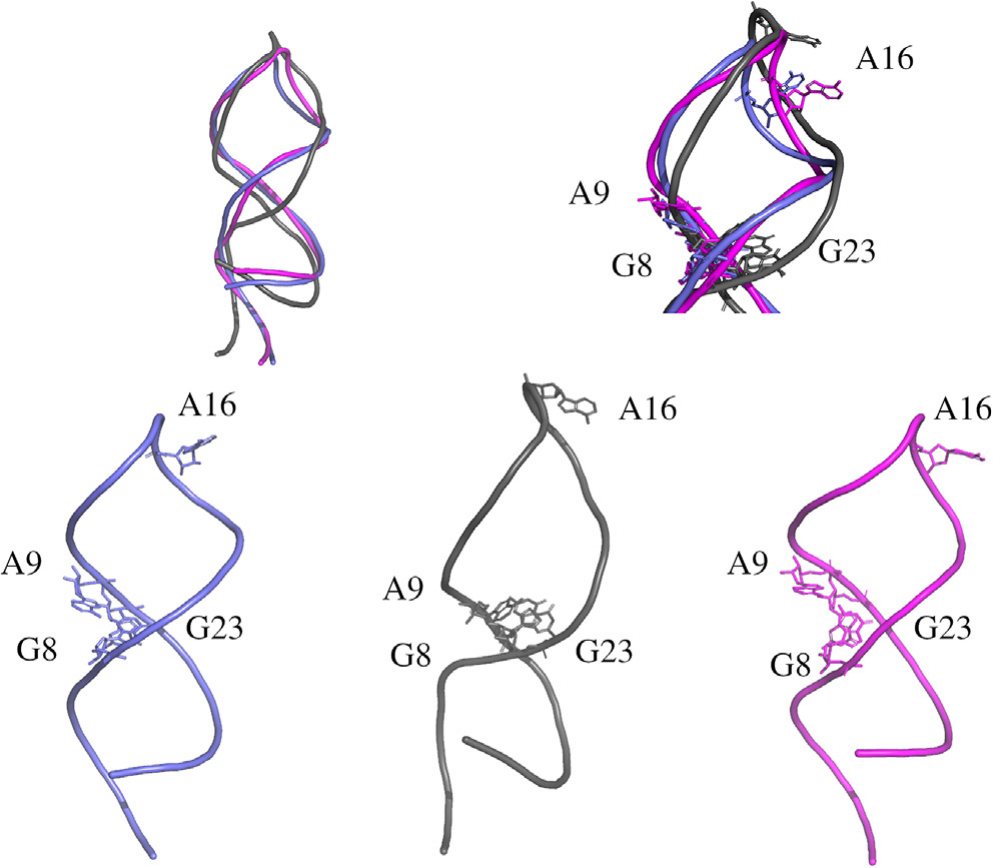
NF-kB aptamer key interactions Top: Global (left) and zoomed (right) views of the superimposition of the NF-κB aptamer conformation (magenta) extracted from the REMD simulation on the free (violet) and bound (gray) aptamer state, as solved with X-ray and NMR methods (PDB codes: 1OOA and 2JWV, respectively), the tetraloop A16 and the internal loop G23, G8, and A9 residues are shown in sticks. Bottom: The different models are individually represented using the same color code.

As expected, the highly flexible region corresponding to the tetraloop exhibits more complex behavior. Indeed, although the structure of this region that emerged from the REMD is frequently divergent from both the apo and bound state (Figure 5), the inspection of the structure ensemble indicates the presence of conformers that closely resemble the tetraloop state observed in the free state of the aptamer (Figure 6). Similarly, the analysis of other indicators such as the inter-base distances or the location of the base A16 indicates that free-like states for this high-mobile region are present in the trajectory despite the bound state used as a starting model in the simulation (Figure 6).

Collectively, these data indicate that for well-structured regions such as the internal loop, the simulations well predict the transition of the aptamer structure from the bound to the free conformation. For the tetraloop region whose extreme mobility has been experimentally demonstrated, the simulation is still able to capture in some of the trajectory structures the features of the free and relaxed state.

## Discussion

RNA tertiary folding is a slow and hierarchical multi-step process occurring on a millisecond timescale or longer, whose description at atomic level is rather inaccessible by either experimental or theoretical methods.^15^⁠ Initial secondary structure formation is followed by slower conformational searches among multiple sub-states of RNA tertiary motifs.^12^⁠ The fast exchange rate among RNA states represents a major obstacle to the discovery and to the effective characterization of the diverse RNA conformations that frequently constitute a basin of distinct functional states through standard NMR or X-ray methods.^16^ Flexibility featuring RNAs is crucial for functionalities and targeting but a complete description of conformational space of RNA molecules of medium size remains unreachable by the current availability of plain molecular dynamic simulation time. Recently, several enhanced theoretical methods were proposed to follow the kinetic of RNA folding and are not only limited to RNAs modest in size.^15^^,^^16^^,^^17^⁠ In 2019, Cheng and coworkers applied a 2D REMD incorporating secondary structure information to describe the RNA folding of four representative RNAs, from 24 to 68 residues in length.^18^⁠ In the same year, Bottaro et al. predicted the structure and dynamics of five RNA stem-loops in the 5′-UTR of SARS-CoV-2 by extensive enhanced sampling techniques of atomistic molecular dynamics simulations.^19^ MD provides insights on the feasibility of RNA motif manipulation in maintaining a peculiar fold, investigating the role of crucial interactions in holding the overall architecture, a key aspect for the rational design of these molecules.^7^^,^^20^^,^^21^

This intricate scenario has a direct impact on the development of new RNA-based molecules with therapeutic potential. Indeed, although the application of computational methods in driving the design and the optimization of new therapeutic agents has been demonstrated for many classes of chemical compounds, their impact on the development of new drugs based on nucleic acids has been rather limited. The paucity of structural and dynamic data on nucleic acids, and, in particular on RNAs, makes the definition of computationally based protocols difficult to achieve.

In this framework, we used here REMD simulations to increase the conformational sampling along the trajectories of two DIR2 aptamer forms, the native parental form (DIR2wt) and a variant embodying a Fab3-6 binding motif (DIR2fab) that was ad hoc inserted into the aptamer sequence to facilitate its structural characterization.^11^⁠ Indeed, the complexation of the aptamer with the Fab favored the crystallographic analysis and provided a three-dimensional structure of the aptamer, although bound to the Fab. The DIR2 aptamer as determined by Piccirilli et al. (pdb code 6db9) folds in a fork-like architecture with a helix and two stem-loops oriented parallel to each other. Here, we performed an extensive conformational sampling of the two DIR2 aptamer variants (DIR2wt and DIR2fab) to measure the effect on the overall conformational space upon the insertion of the Fab binding motif within the RNA sequence. The MD simulation analysis clearly suggests that both variants retain the overall structure exhibited in the complex with the Fab. Nevertheless, present data indicate that DIR2wt and DIR2fab are endowed with remarkable flexibility. This finding well agrees with the experimental difficulties encountered in crystallizing DIR2 as an individual entity. In this general framework, a deep inspection of the trajectory structures also highlights small but significant differences in the overall flexibility of the two aptamers. In particular, the insertion of extra bases in the L2 loop of DIR2fab deputed to the Fab anchoring leads to a significant increase of the local flexibility that affects also some distant regions. Notably, these changes only marginally affect the binding site of fluorophores, in line with the similar affinity of these compounds for these two forms. The analysis of the interaction network of the two variants also provides a structural explanation for the increased flexibility of the aptamer bearing the insertion. Indeed, the modification of the L2 loop leads to the loss of a key intra-motif base connection (U24-G52) that is present in the parental aptamer and is missed in the DIR2fab (Figure 3). The loss of this contact loosens the interactions of the L2 loop with the rest of the aptamer and produces an overall increase of the flexibility. In conclusion, the present findings indicate that the modular structure of RNA molecules allows significant insertions or deletions with major structural reorganization. However, these changes may have a significant impact on the RNA plasticity. Therefore, the functionalization of aptamers with Fab binding motifs is certainly an effective strategy to gain structural information on these intricate molecules. On the other hand, to achieve a full understanding of the behavior of these molecules, once obtained, these static data should be complemented by extensive sampling analyses as those illustrated in the present work. In general, important correlations between the tertiary folds and the RNA sequence that are inaccessible by the most diffuse experimental methods can emerge by applying the presented theoretical approach and can guide the optimization and the rational design of RNA-based molecules.^22^^,^^23^

Moreover, we explored the ability of this approach to deal with the identification of the structural basis of protein-aptamer recognition, which is a fundamental issue for the optimization of the effectiveness of these RNA molecules. Although the structure of several dozens of protein-aptamer complexes has been experimentally determined,^24^ in the vast majority of the cases no information has been collected on the intrinsic structural/dynamic properties of the aptamer,^24^^,^^25^ a crucial step for a full understanding of the determinants of the binding and recognition. By using an RNA aptamer specifically selected to target the transcription factor NF-κB, we show that the approach here illustrated can appropriately reproduce the main structural and dynamic properties of the free aptamer starting from the conformation it adopts in the complex with the protein thus providing information on both the contributions of the aptamer preorganization that either of the induced fit events provides to the binding.^13^^,^^14^

In this context, it is important to note that for most therapeutic purposes, the initially identified aptamers are frequently heavily modified for several reasons.^26^^,^^27^ Indeed, they can be truncated to reduce synthesis costs, modified to increase nuclease resistance, rigidified to allow structural characterizations, and conjugated to different chemical entities to reduce renal filtration.^27^ Therefore, the availability of computational tools, such as those described in the present paper, that provide rapid and reliable descriptions of the structural and dynamic responses of RNA molecules in response to changes in the local environment, i.e., dissociation from the partner, or as a consequence of sequence^13^ modifications represent a powerful and valuable tool for the optimization of drug candidates. Moreover, effective computational characterizations of the structural and dynamic properties of RNA molecules may be crucial for either the *a priori* screening of many variants of hit compounds to reduce drug development costs or for the rational design of nucleic acid therapeutics.

## Materials and methods

The three-dimensional structure of the DIR2fab and NF-κB aptamers was extracted by the pdb solved structures, downloaded from the Protein Data Bank database (PDB: 6DB9 and 1OOA, respectively).^11^^,^^14^

MD simulations were run using the amber14sb force field including the most recent parameters properly designed to describe RNA systems implemented for GROMACS 2020/5.0.5.^22^^,^^23^^,^^28^^,^^29^^,^^30^⁠ Both the systems were solvated in an octahedron box using the TIP3P water model 40 with a 1.1-nm distance to the border of the molecule, simulating standard biological conditions by considering a 150-mM KCl concentration and additional ions to neutralize. Electrostatic interactions were treated using the particle mesh Ewald method and Berendsen algorithm to control temperature and pressure,^31^^,^^32^ following the indications dictated by the ABC consortium (https://bisi.ibcp.fr/ABC/Protocol.html) and previous protocols applied on nucleic acids and derivatives.^7^^,^^33^^,^^34^⁠ In all the systems, waters were first relaxed by energy minimization and 10 ps of simulations at 300 K, restraining the RNA atomic positions with a harmonic potential. Then, the systems were heated up gradually to 300 K in a six-step phase starting from 50 K. Finally, the equilibrated systems were subjected to 64 T-REMD simulations with a temperature distribution ranging from 300 to 385 K and an exchange trial between adjacent replicas every 1,000 steps of 2 fs. According to the protocol proposed by Qi et al.,^12^ 64 parallel simulations were run in NPT standard conditions for 40 ns without restraints. The temperatures of each replica were defined using the http://virtualchemistry.org/remd-temperature-generator/ ^35^ in SuppREMD. The convergence analysis for T-REMD runs shows replica index and temperature exchange plot along the simulation time for both the aptamers (Figure S6). GROMACS,^28^^,^^29^⁠ VMD,^36^ and Pymol packages^37^⁠ were used to analyze the 300-K run trajectories. Clustering analyses of the both MD simulations were performed to extract representative conformations using the gromos method with the algorithm described by Daura et al.^38^ For each cluster, the structure exhibiting the lowest RMSD relative to all the other members of the cluster was selected as representative. The secondary structure pictures depicted in Figure 1 were created by using RiboSketch web tool.^39^

## Data and code availability

The data that support the findings of this study are available from the corresponding author, Ida Autiero, at the https://github.com/idaaut/MolTher23 GitHub repository address.

## Supplemental information

Document S1. Figures S1–S8

Document S2. Article plus supplemental information
