# Supplementary material for: Enhanced molecular dynamic simulation studies unravel long-range effects caused by sequence variations and partner binding in RNA aptamers
Source: Mol Ther Nucleic Acids. 2023 Sep 29;34:102039. doi: 10.1016/j.omtn.2023.102039 (PMC10585333; doi:10.1016/j.omtn.2023.102039)
Supplement: Document S1. Figures S1–S8 [file mmc1.pdf]

OMTN, Volume *34*

Supplemental information

Enhanced molecular dynamic simulation studies unravel long-range effects caused by sequence variations and partner binding in RNA aptamers

Ida Autiero and Luigi Vitagliano

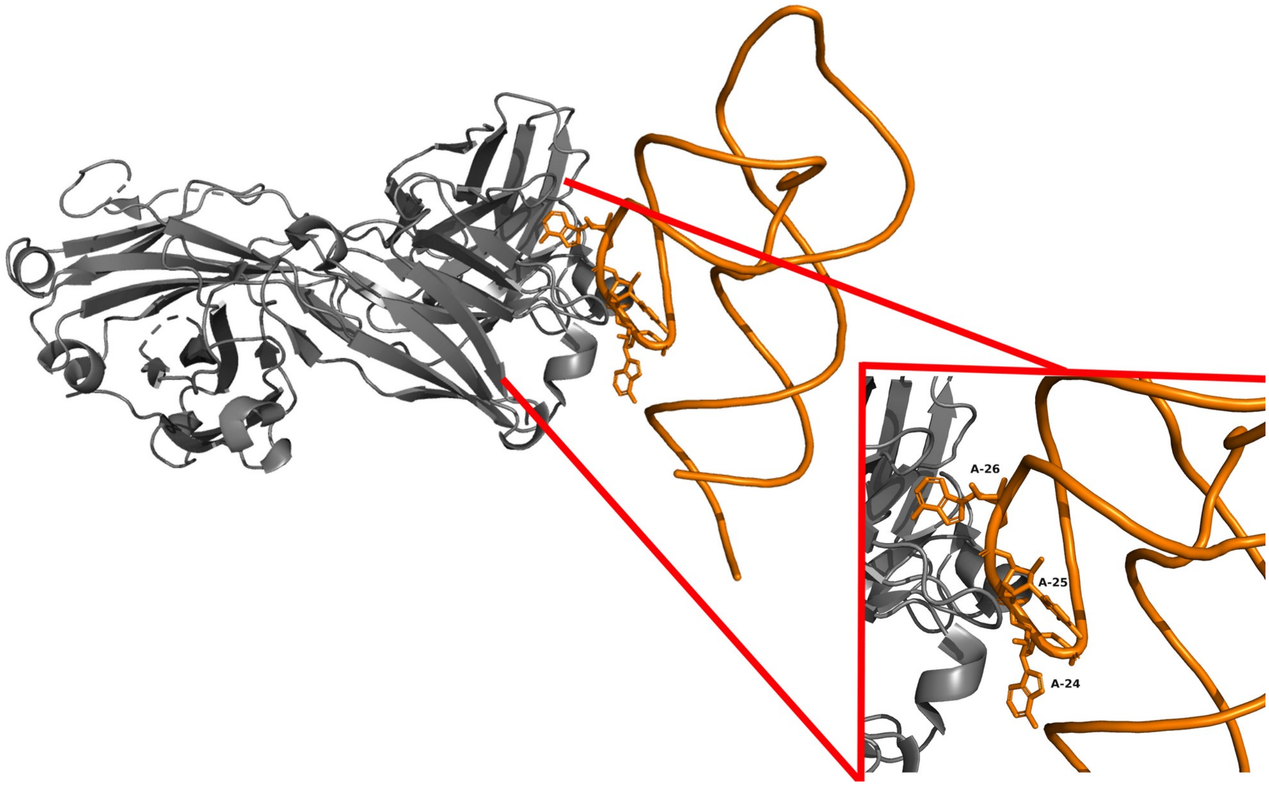

Figure S1. Cartoon representation of the crystal structure of the DIR2s aptamer (in light orange) in complex with Fab BL3-6 (gray) as solved and deposited with the pdb code: 6DB9[2]. The residues belonging to the Fab binding site are labeled and represented by orange sticks in the red zoom panel.

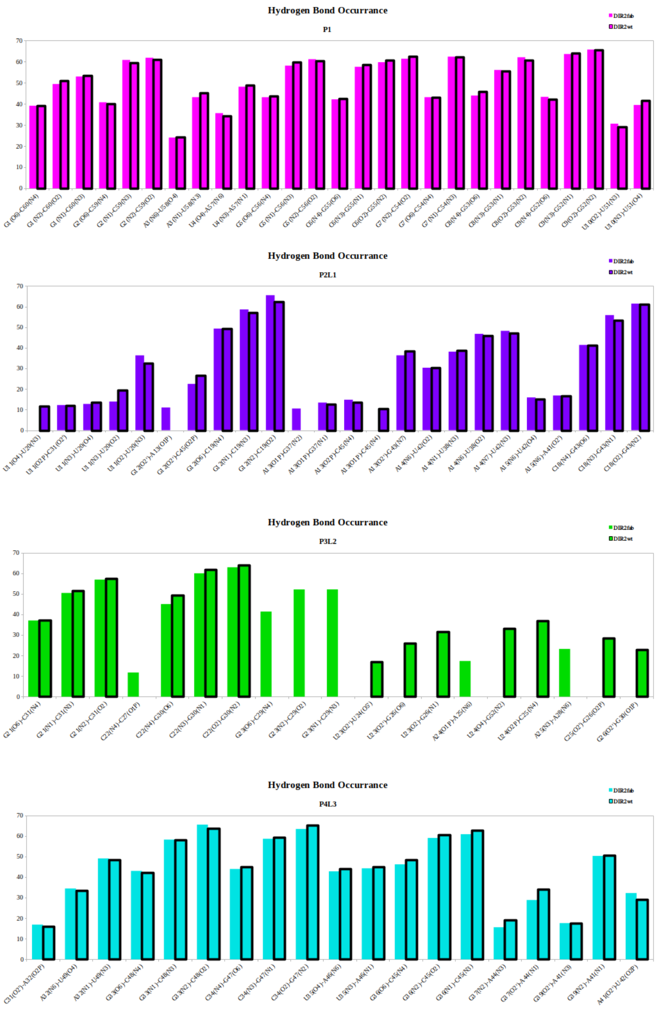

Figure S2. The percentage of occurrence of persistent (> 10% of the run frames) hydrogen bonds along the DIR2fab and DIR2wt simulations are reported in bar plots. The pairs are colored following the scheme of secondary structure motifs, magenta:P1, violet: P2L1, green:P3L2 and cyan: P4L2.

MOTIF COM DISTANCE

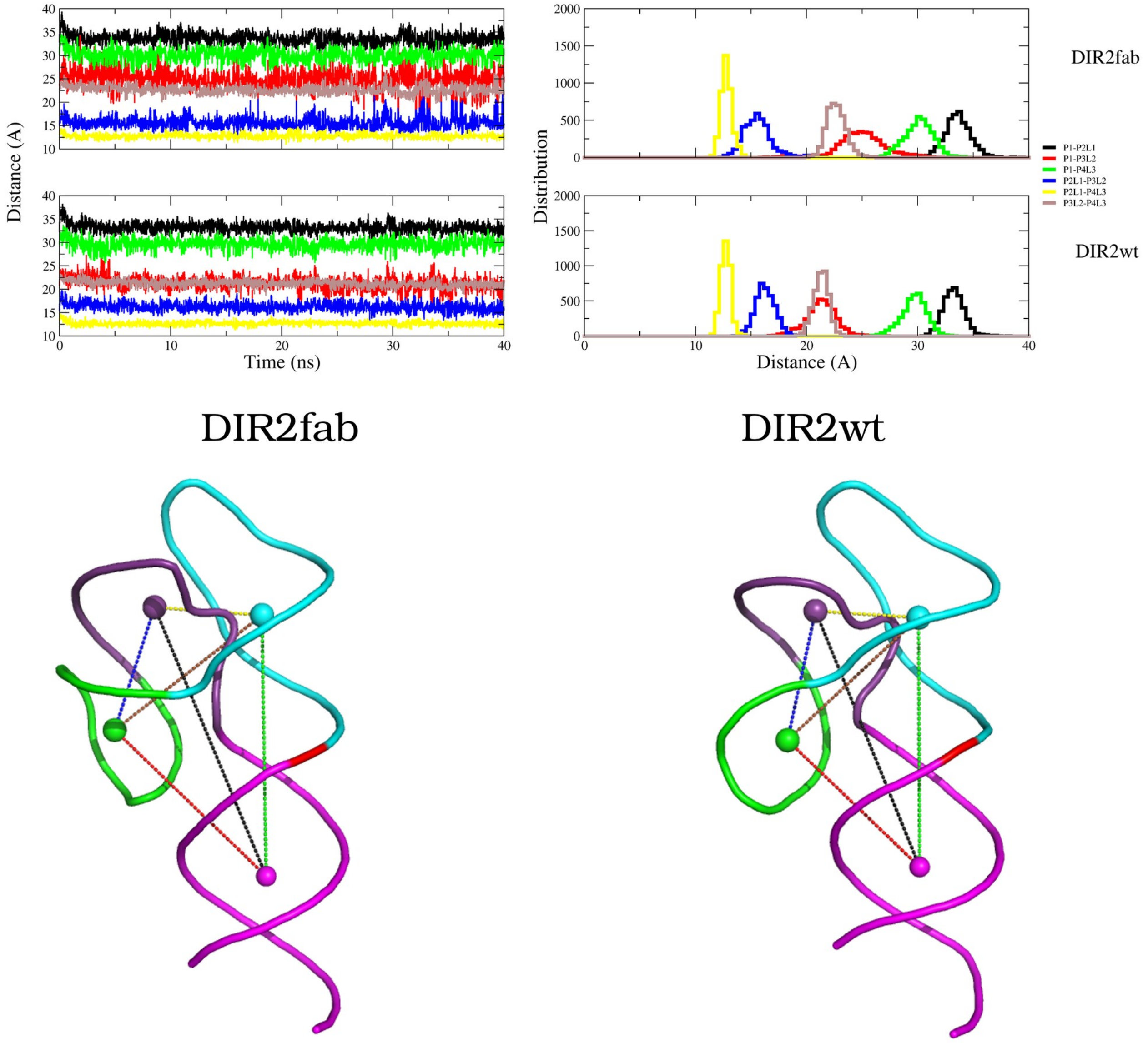

Figure S3. Distances between the secondary structure motifs center of mass expressed as distances along the simulation time (*Left panels*) and as distribution of the distance values (*right panels*). *Bottom panel*: cartoon representation of the representative structures of the DIR2fab (left) and DIR3wt (right) aptamers with the indication of the center of mass of each secondary structure motif considered for the analysis and colored by the secondary structure motif color scheme (magenta:P1, violet: P2L1, green:P3L2 and cyan: P4L2).

DIHEDRAL GLYCOSIDIC ANGLE

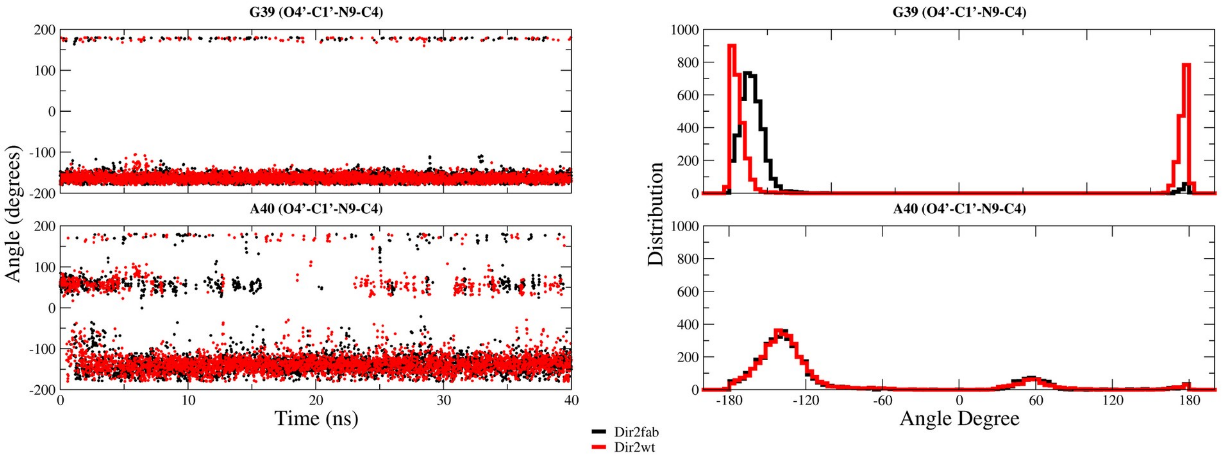

Figure S4. Dihedral glycosid angle of the G39 (*top panels*) and A40 (*bottom panels*) expressed as angle along the simulation time (l*eft*) and as distribution of the angle values (*right*).

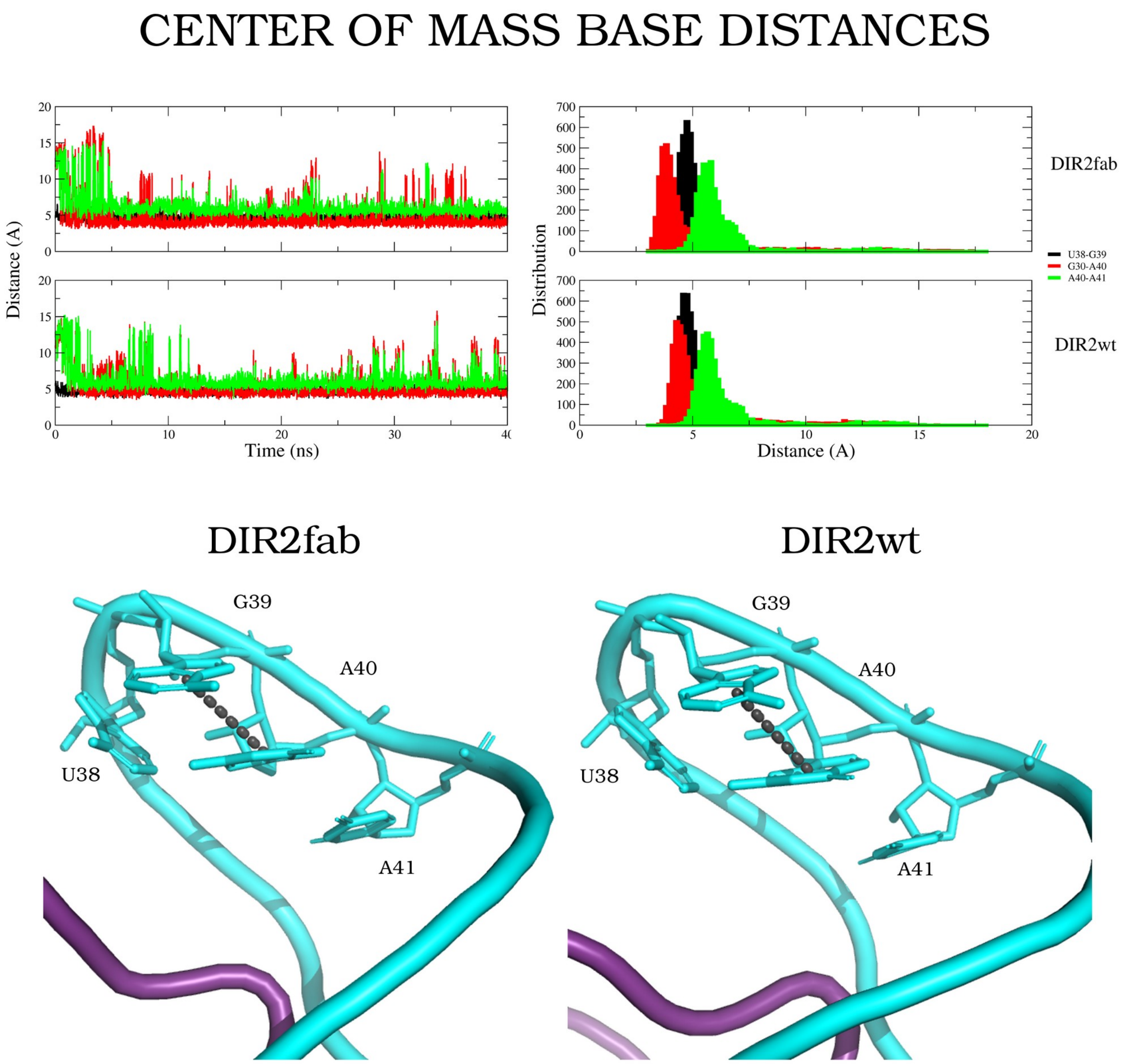

Figure S5. Distances of the center of mass of the G39 and A40 base atoms with the respect to the previous and the succeeding residues (38-39, 39-40 and 40-41 pairs) expressed as distances along the simulation time (*Left panels*) and as distribution of the distance values (*right panels*). *Bottom panel*: representative structures of the DIR2fab (left) and DIR3wt (right) aptamers with a zoom representation of the residues considered in the analysis.

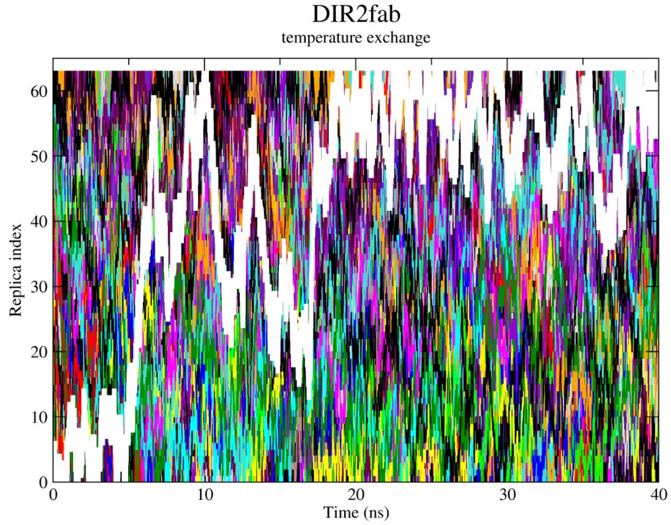

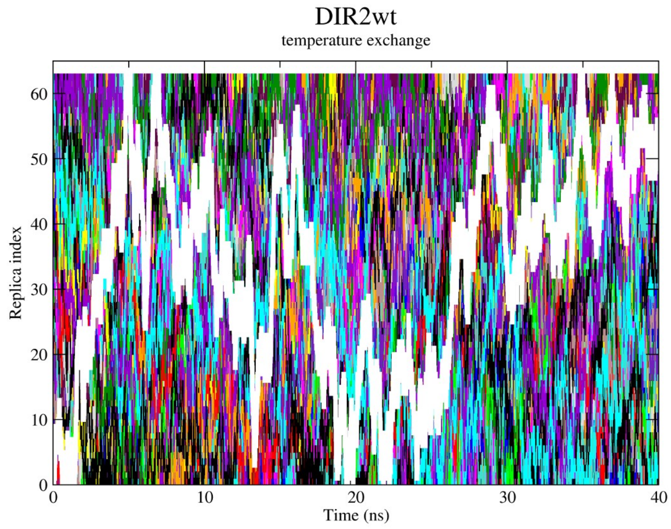

Figure S6. Convergence check of the REMD DIR2fab (top) and DIR2wt (bottom) runs. Exchanges of replicas colored by following temperature indices of 64 runs, those starting at 300K are indicated with tick white line.

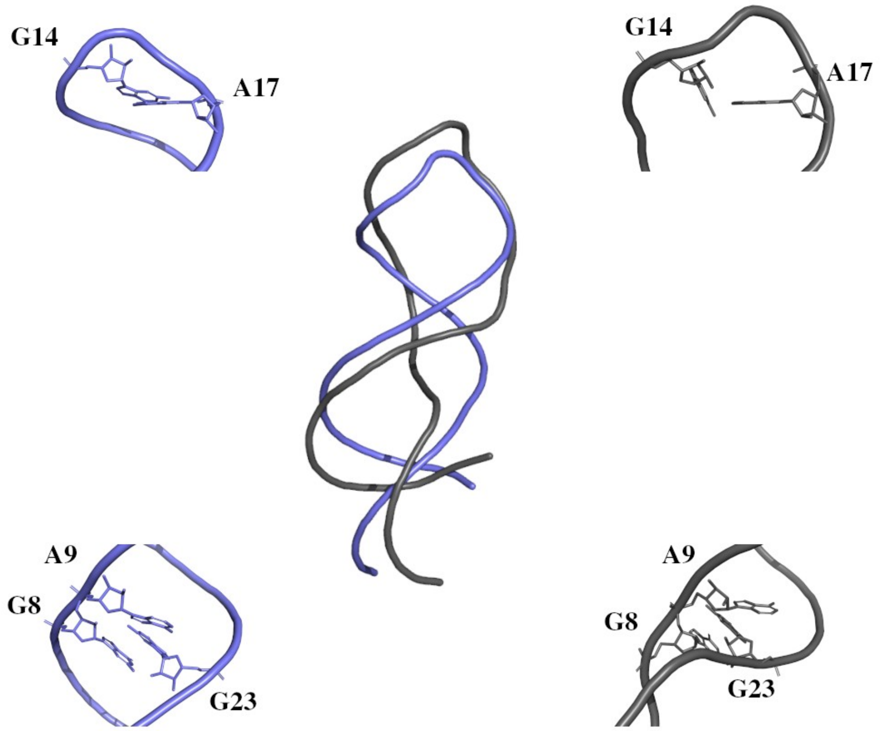

Figure S7. Global and local views of the unbound (violet) and bound (gray) states of the NF-kB aptamer as emerged from the experimental studies (PDB codes: 2JWV and 1OOA, respectively). The zoomed windows represent the specific side chains of tetraloop termini (G14 and A17) and tetraloop (G8, A9 and G23) residues responsible of the main differences between the unbound and bound aptamer states.

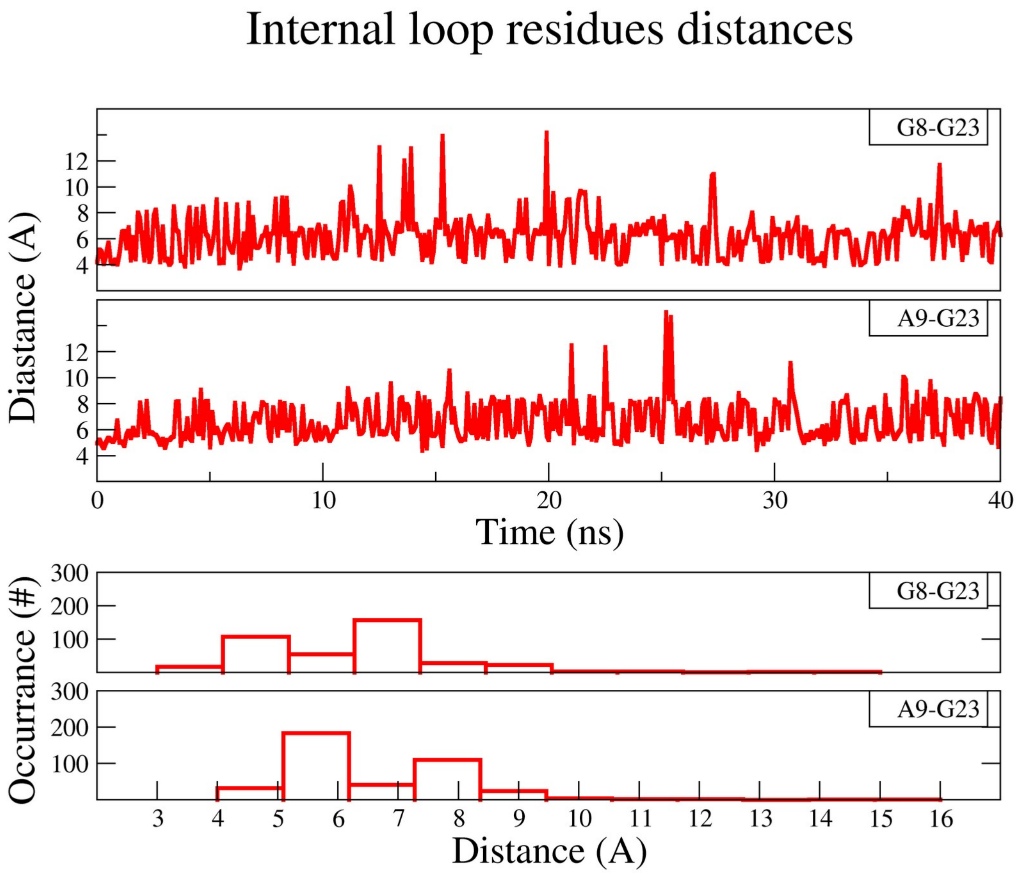

Figure S8. Distances between the internal loop residues along the REMD simulations. In the experimentally solved states, the G8(N9)-G23(N9) and A9(N9)-G23(N9) distances were: 6.4 and 6.7A in the NF-kB-free and 4.7 and 3.8 in NF-kB-bound experimental structures, respectively.
